# Obesity and Brain Function: The Brain–Body Crosstalk

**DOI:** 10.3390/medicina56100499

**Published:** 2020-09-24

**Authors:** Sophia X. Sui, Julie A. Pasco

**Affiliations:** 1 IMPACT—The Institute for Mental and Physical Health and Clinical Translation, School of Medicine, Deakin University, Geelong, VIC 3220, Australia; julie.pasco@deakin.edu.au; 2 Department of Medicine-Western Health, The University of Melbourne, St Albans, VIC 3021, Australia; 3 Barwon Health, Geelong, VIC 3220, Australia; 4 Department of Epidemiology and Preventive Medicine, Monash University, Melbourne, VIC 3181, Australia

**Keywords:** obesity, cognitive function, brain function, BDNF, leptin, inflammation

## Abstract

Dementia comprises a wide range of progressive and acquired neurocognitive disorders. Obesity, defined as excessive body fat tissue, is a common health issue world-wide and a risk factor for dementia. The adverse effects of obesity on the brain and the central nervous system have been the subject of considerable research. The aim of this review is to explore the available evidence in the field of body–brain crosstalk focusing on obesity and brain function, to identify the major research measurements and methodologies used in the field, to discuss the potential risk factors and biological mechanisms, and to identify the research gap as a precursor to systematic reviews and empirical studies in more focused topics related to the obesity–brain relationship. To conclude, obesity appears to be associated with reduced brain function. However, obesity is a complex health condition, while the human brain is the most complicated organ, so research in this area is difficult. Inconsistency in definitions and measurement techniques detract from the literature on brain–body relationships. Advanced techniques developed in recent years are capable of improving investigations of this relationship.

## 1. Introduction

Obesity is characterised by an excessive accumulation of adipose tissue [1]. Obesity is often identified by body mass index (BMI, kg/m^2^), which is calculated as weight (kg) divided by height squared (m^2^); there are typically three categories: normal weight (BMI 18.5–24.9 kg/m^2^), overweight (BMI 25.0–29.9 kg/m^2^), and obese (BMI ≥ 30.0 kg/m^2^) [2]. However, the use of BMI as a marker of obesity is flawed as it does not take into account differences in body composition or body fat distribution. Obesity affects a high proportion of the global population [3], and it is estimated that about two thirds of Australian adults are overweight or obese [4].

As a major cause of premature death and illness, obesity is associated with an increase in the risk of chronic diseases, such as type 2 diabetes and cancer [5], and other health problems. Around 70% of the obese population has complicated obesity, defined as obesity with metabolic diseases or the metabolic syndrome, which includes insulin resistance, hypertension, abdominal obesity and inflammation, while the remainder have uncomplicated obesity, defined as obesity with no apparent metabolic abnormalities [6]. One of the possible mechanisms contributing to uncomplicated obesity is leptin deficiency, which can impair appetite, thus leading to increasing body weight in the absence of other metabolic abnormalities [7]. As unhealthy diet and insufficient physical exercise also contribute to obesity, obesity (especially uncomplicated obesity) is potentially reversible by physical and nutritional interventions; however, obesity can also be characterised as a persistent condition [8].

The evidence demonstrates that obesity is associated with cognitive deficits, which refers to diminished or impaired mental and/or intellectual functioning. Obesity has been linked with functional and structural brain change in neuroimaging studies and is associated with a risk of developing Alzheimer’s disease (AD) [9]. The underlying mechanism is that adipokines, such as interleukin (IL-6), tumour necrosis factor alpha (TNF-α), and leptin, are able to cross the blood–brain barrier and affect the brain [10,11,12]. In this review, we critically examine the relationship between obesity and the brain. We emphasise obesity-related cognitive impairment and dementia, but we also address the more subtle changes in brain structure and functioning in obesity. We also discuss pre-clinical studies and epidemiological studies that reveal the biological mechanisms underlying obesity-associated brain dysfunction or risk factors related to this relationship. Finally, we describe methods for detecting parallel and subtle changes in body composition and the brain that might have utility for future studies.

## 2. Obesity, Weight Loss, and Neurocognitive Function

Cognitive function is defined as an “intellectual or mental process whereby an organism obtains knowledge” [13]. Cognitive function is commonly considered overall or located in specific domains. The specific cognitive domains include memory, learning, attention/executive function, information processing, psychomotor function, and language [13,14]. Substantial epidemiological evidence suggests that obesity is associated with poor cognitive function. However, we acknowledge that neither obesity nor cognitive function is a unidimensional construct, which means that the severity and progression of obesity-associated cognitive decline/impairment may vary, depending on the type and duration of obesity and its related comorbidities.

In a cross-sectional study in Canada [15] published in 2009, 207 participants aged ≥18 years were recruited from the community; of these, 56 were identified as obese and 24 as overweight, using BMI. Poor cognitive function was assessed by the Clock Drawing Test and the Trail Making Test in combination with an executive function assessment. Briefly, participants were instructed to draw in the numbers of a clock face in the Clock Drawing task; participants were scored by two researchers according to accuracy. In the Trail Making Test (part A), participants were asked to connect 25 circles on a piece of paper in an ascending sequence as fast as possible; the maximum time allowed was 90 s. In Part B, participants were asked to draw a line alternating between numbers and letters in ascending sequences. A faster speed indicated better performance in both tasks. Multivariable analysis showed that, compared to those who were not obese, obese participants were almost fourfold more likely to exhibit poor executive function. Additionally, metabolic syndrome was associated with poor cognitive performance.

A systematic literature review by Prickett et al., (2015) [16] of 17 cross-sectional studies, examined the relationship between obesity independent of comorbidities and cognitive function in adults aged 18–65 years. Their analysis suggested that obesity was associated with cognitive impairment in all tested domains, including memory, attention and decision-making. However, the authors concluded that there was insufficient evidence that obesity was independently associated with cognitive impairment in middle-aged adults, due to methodological limitations related to controlling for comorbid conditions.

Elias et al., (2005) [17] examined the effect of obesity independent of diabetes mellitus (non-insulin-dependent) on cognitive performance in an 18-year longitudinal study, involving 1423 participants (61% women) who were free of dementia, stroke, and cardiovascular disease at the time of cognitive assessment. This study reported an effect of obesity on cognitive function in men, but not in women. Another study in the United States of America (USA) examined the long-term bidirectional relationship between obesity and executive function or episodic memory [18]. This study included 2652 adults aged 33–84 years (55% women) who were followed up after nine years. BMI was self-reported, while cognitive function was assessed using the Brief Test of Adult Cognition by Telephone that focused on executive function (e.g., performance including backward digit span, categorical fluency, backward counting) and episodic memory (e.g., word list recall and delayed word list recall). The study showed that greater obesity at baseline was associated with a decline in episodic memory at the follow-up stage, while better executive function at baseline was associated with a reduction in obesity at follow-up, suggesting a bidirectional relationship between obesity and cognitive function.

Findings to date provide less support for the contention that obesity has an impact on general cognitive performance, when measured by general cognitive tests or general intelligence quotient (IQ) performance, while showing that obesity is associated with poor performance in cognitive domains including executive function, memory, and information processing. Life factors, such as age, socio-economic status or metabolic diseases, which directly contribute to cognitive function or interact with obesity, may reduce cognitive performance in each domain [19]. Additionally, the use of different cognitive assessments appears to contribute to these inconsistencies. A review published in 2011 [20] assessed the associations between obesity and cognitive function across the lifespan and suggested that weight gain was associated with reduced executive function, and that the underlying mechanisms include low-grade systemic inflammation, elevated lipids and/or insulin resistance. However, associations between obesity and other cognitive domains or general cognitive function were mixed.

The direction of the relationship between obesity and cognitive function is less clear in the elderly. In the review by Smith et al., (2011) [20], the relationship between obesity and cognitive function in old age was the inverse of the patterns observed in younger people. In particular, unintentional weight loss in older adults was linked with poor cognitive outcomes [21]. Ageing generally includes a decline in skeletal muscle mass and an increase in body fat, sometimes with no apparent change in overall body weight or BMI. When sarcopenia, a condition of loss of muscle mass and function with advancing age, occurs in the face of excessive fat accumulation, the condition is known as sarcopenic obesity, and is more common with increasing age and adiposity [22]. Wang et al., (2019) [23] examined the associations between sarcopenic obesity and cognitive impairment in 948 community-dwelling Chinese people aged 60–92 years. Cognitive function was assessed using the Mini-Mental State Examination (on which a score ≤ 24 suggests mild dementia), whole body composition was estimated using bioimpedance analysis, and the participants were separated into four groups: sarcopenic obese, sarcopenic, obese, and normal, determined by their levels of muscle mass and fat mass. Results revealed that the sarcopenic obese group and the obese group were 2.5 times or 2.1 times more likely (respectively) to have cognitive impairment, after adjusting for confounders.

Research into the effects of involuntary weight loss in the elderly and intentional weight loss provides a different story. Siervo et al., (2011) [24] conducted a systematic review and meta-analysis to examine the associations between intentional weight loss and cognitive function (memory and attention) in overweight and obese adults. This analysis included 12 studies, seven of which were randomised controlled trials. In this study, weight loss was associated with an improvement in cognitive performance in memory attention/executive functioning in people who were obese but not overweight. Similarly, Veronese et al., (2017) [25] conducted a meta-analysis of randomised controlled studies (*n* = 7) and longitudinal studies (*n* = 13) to examine how voluntary weight loss affected cognitive domains (memory, attention, executive function, language, and motor speed) in obese and overweight people. This study involved 1019 participants, and the interventions included diet, physical activity and bariatric surgery. The findings were that weight loss was associated with improved attention and memory in both types of study design and was associated with improved executive function in longitudinal studies and with improved language in trials.

## 3. Obesity and Dementia

Dementia encompasses a wide range of progressive and acquired neurocognitive disorders. The core feature is a loss of intellectual abilities severe enough to disturb social and occupational functioning. Dysfunction involves behaviours in multiple cognitive domains, such as executive function, complex attention, learning and memory, language, perceptual-motor, and social cognition [26]. AD is the major cause of dementia, accounting for 50–70% of dementia cases. It is a progressive, degenerative disorder involving loss of memory, thinking and language skills, and behavioural changes [27].

Several studies have shown that being obese or even overweight is associated with an increased risk of AD. An 18-year follow-up study in Germany by Gustafson et al., (2003) [28] found that women with dementia aged 79–88 years were overweight and had a higher average BMI at ages 70, 75 and 79 years than women without dementia. These findings suggest that obesity is associated with a greater likelihood of AD, particularly in women. Given that a large volume of evidence points to an association between obesity and cognitive disorders coinciding with ageing, some researchers have theorised that obesity affects cognitive decline by accelerating ageing. However, Gustafson et al.’s (2003) study did not include younger participants, so this hypothesis could not be tested.

Fitzpatrick et al., (2009) [29] noted an obesity paradox, which is that being either underweight or obese is associated with a greater risk of dementia in older populations. Rapid weight loss in older obese people was found to be a marker of dementia in later life, possibly because weight loss in old individuals can be the result of neuropathological diseases or normal cognitive decline [29]. In a systematic literature review and meta-analysis of 21 longitudinal studies, Pedditzi et al., (2016) [30] examined overweight or obesity as a risk factor for incident dementia in mid-life and later life. Obese people aged <65 years were 1.4 times more likely to have incident dementia, but those aged ≥65 years were less likely to develop dementia within 2 years of follow-up. A longitudinal study by Singh-Manoux [31] confirmed a similar pattern. This study examined the association between obesity and incident dementia in 10,308 adults (67% men) aged 35–55 years at baseline and followed them for 30 years. BMI was assessed six times, and 329 people were ultimately diagnosed with dementia. Compared with the people who were cognitively normal, BMI in people with incident dementia was lower eight years before diagnosis, but higher 16–28 years before diagnosis.

The fact that some obesity measurement methods cannot capture body composition changes in later life contributes to these inconsistent conclusions [4]. The findings appear to depend on the way obesity is recognised. One study reported a positive association between obesity and cognitive impairment in elderly participants when using waist circumference to define obesity but found the opposite when applying the BMI method in the same cohort [32]. This suggested that cognitive impairment was more likely associated with central (or truncal), rather than overall, accumulation of fat. To date, most empirical studies have used BMI as a surrogate measure of obesity; however, other measurements, such as waist circumference and waist-to-hip ratio, are considered better for characterising central obesity, because BMI alone may not account for differences in fat storage locations. It is also of note that, as BMI represents weight indexed to height, it does not capture differences in the relative contributions of muscle and fat to body weight.

## 4. Obesity and Functional and Structural Brain Changes

Changes in brain volume and density are often reported as measures of structural brain changes. These structural changes have been reported in obese populations, especially in elderly people, and growing evidence suggests that obesity is associated with regional structural alterations [33]. A tensor-based morphometry study [34] of cognitively healthy obese older individuals reported a reduced volume in the frontal lobes, anterior cingulate gyrus, hippocampus, and thalamus. Similarly, high BMI has been negatively associated with the integrity of the frontal lobes in middle-aged adults [35] and the elderly [36]. In addition, obesity has been associated with global brain structural change, for example, overall cerebral atrophy in grey matter and white matter volume [34].

Yau et al., (2014) [37] detected signs of cortical thinning in specific brain regions and reduced microstructural integrity in white matter tracts in obese adolescents, and associated overall lower academic performance with structural impairments, but obese youths performed no worse than non-obese youths in cognitive tests in this study. While associations between brain structural alterations and cognitive performance cannot inform causality, smaller sizes and volumes indicate neuronal loss, and therefore it is biologically plausible to link these changes to poor cognitive performance. However, why brain structural changes did not determine poor cognitive outcomes in this study is unclear. Perhaps changes in the brain become evident in obese adolescents, but the impact of these changes on cognition are not manifested until later in life. This puzzle may be explored using advanced brain imaging techniques that are more likely to detect structural micro-changes that are not translated into immediate cognitive deficits in early stages of decline. Therefore, it is worthwhile combining physiological assessment with cognitive assessments to explore the impact of obesity on brain function.

Opstal et al., (2019) [38] examined the impact of weight loss (prolonged fasting) on brain activity in obese people. This study included 14 participants (two men) who were categorised as obese by BMI. Brain imaging data were collected after an overnight fast, a 48 h fast and an 8 week intervention for weight loss using whole-brain resting-state functional magnetic resonance imaging (MRI). The authors reported that the weight loss intervention decreased activation in the parts of the brain involved in salience, sensory motor, and executive control, suggesting a relationship between obesity-related changes in neurological activity and weight loss. Recently, a study by Opel et al., 2020 [39] conducted a mega-analysis investigating brain structure abnormalities and obesity and also considered the effect factors of age, genetic risk, and psychiatric disorders. This study examined the relationship between obesity (BMI > 30 kg/m^2^) and brain structure involving in 6420 participants. Findings revealed that obesity was significantly associated with cortical and subcortical abnormalities independently, especially in lower temporo-frontal cortical thickness. A higher polygenic risk score and age played an interaction role in cortical thickness.

## 5. Risk Factors

### 5.1. Childhood Maltreatment

Tan et al., 2014 [40] conducted a meta-analysis of 41 studies including 285 participants and revealed that childhood maltreatment was associated with an elevated risk of developing obesity over the life span. This association was consistent, despite differences in study design, measures, definitions, and confounding or mediating variables considered (e.g., socioeconomic status, current smoking, alcohol intake, or physical activity). A stressful psychosocial experience in childhood, such as emotional neglect or depression, was also identified as a risk factor for obesity. Furthermore, there were modifying effects of gender and ethnicity. Another systematic review of 11 prospective cohort studies [41] examined the causal associations between childhood maltreatment and cognition in prospective studies. Participants from these studies were mostly children or adolescents who were followed up over several years. The results revealed that maltreatment was associated with cognitive function in nine domains after controlling for potential confounders. Furthermore, Young-Southward et al., 2020 conducted a systematic review of 31 studies investigating the causal relationship between maltreatment and cognition in children under 12 years old. The results suggested that the strength of the causal relationship between maltreatment and lower IQ and cognitive development was determined by the duration and timing of maltreatment. To note, findings were less robust for children from non-institutionalised organisations compared with those who were institutionalised.

In a study involving 547 participants with depressive disorder and 670 healthy controls (mean age 34.7 years (SD 13.2); 37.6% male), Goltermann et al., 2020 [42] examined the relationship between childhood maltreatment and cognition in several domains, and also estimated potential confounders (genetic and environmental factors) and nature–nurture interactions. The assessed cognitive domains included working memory, executive functioning, processing speed, attention, memory, and verbal intelligence. Results revealed that each cognitive domain was independently associated with childhood maltreatment, depression, parental education, and polygenic scores for depression and educational attainment.

### 5.2. Biological Pathways

Brain-derived neurotropic factor (BDNF) is a candidate for the mechanism linking obesity and adverse brain function. Imaging of brain glucose by positron emission tomography is a standard method for assessing brain metabolism in vivo. Lozzo et al.’s (2019) review [43] assessed studies that employed this technique in attempts to reveal the underlying mechanisms that link obesity and cognitive dysfunction. They concluded that brain hypermetabolism is associated with cognitive control for food rewards, low BDNF levels, and insulin resistance. Obesity is associated with reduced levels of BDNF or reduced BDNF signalling, which are associated with deficits in neuronal and behavioural plasticity [44]. Polymorphisms in the BDNF gene influence activity-related release of BDNF. BDNF levels appear to be influenced by genetics, but evidence also suggests that they are associated with lifestyle and diet. One study observed reductions in BDNF in rats eating a high-fat or high sugar diet [45]. Some evidence was found to suggest an association between BDNF and impaired neuroplasticity and reduced learning and memory. For example, unrestricted access to a high-fat diet in rats increased body weight, decreased BNDF levels in the prefrontal cortex and ventral hippocampus, reduced learning performance, and impaired the control of food intake [46].

An early report demonstrated a cross-sectional association between serum leptin and obesity expressed as percentage body fat [47]. An abnormally high level of leptin (hyperleptinemia) plays a role in polygenic obesity, while low leptin (hypoleptinemia) is associated with weight loss from an obese state [48]. Further, cross-sectional data suggest that inefficient leptin signalling is partly associated with changes in hippocampus structure and decreases in memory performance [49]. Lower leptin was found to contribute to poorer memory through its negative effect on hippocampus volume and microstructure [49]. In rodents, leptin deficits are associated with impairments in synaptic signalling [50]. In a genetic obesity animal model with leptin deficits, for example, synaptic plasticity was shown to be impaired in the hippocampus [51]. Rats with leptin deficits exhibit poorer performance in a series of learning tasks, as well as impaired synaptic plasticity in the hippocampus [51]. In humans, Sanborn et al., 2020 [52] examined the association between plasma leptin levels and neuropsychological performance and brain structures (neuroimaging) and explored how BMI plays a role underlying this association in earlier middle-aged adults. This study included 2223 adults (53% female) and found that the association between higher levels of leptin and better verbal memory was observed among the healthy-weight participants but no association was observed among participants who were overweight or obese, suggesting that BMI is an effect modifier of this association. This study suggested that prospective studies in older adults are needed to examine the leptin contribution to AD and other neurodegenerative diseases.

Systemic and central inflammation is associated with obesity and has been linked with impaired synaptic neuroplasticity and cognitive decline [44]. Pro-inflammatory cytokines (e.g., interleukin 1) derived from adipose tissue suppresses neuronal function in the brain, thus impairing synaptic plasticity and cognitive function [53]. For example, a study found that surgical fat transplantation in rats resulted in systemic and central inflammation, which adversely affected synaptic plasticity and cognitive function, suggesting that inflammation mediates the impairment of cognitive and synaptic plasticity [53]. Feinkohl et al., 2020 [54] examined the associations between plasma adipokine concentration (leptin and adiponectin) and cognitive impairment in 669 men and women aged 65 years and over from Germany and the Netherlands. Cognitive performance was measured using neuropsychological tests and the lowest tertile scores of the summary scores of these tests were considered as cognitive impairment. The study found that higher leptin concentrations and a higher leptin/adiponectin ratio were associated with cognitive impairment in the non-obese group only, suggesting that the role of leptin in cognitive impairment with advanced ageing may be limited to non-obese individuals.

## 6. Techniques for Assessing Obesity

There are several techniques for measuring adiposity. Dual energy X-ray absorptiometry (DXA) is widely used for measuring lean mass, fat mass, and body mineral content [55,56]. DXA uses two X-ray beams of different energy levels that are differentially absorbed by bone and soft tissue. The strengths of DXA are its high precision, accuracy and reproducibility, low cost, and low radiation exposure. However, DXA machines are large and not readily portable, project three-dimensional (3D) measurements from two-dimensional images, and require specific technical skills for their operation [55,56]. Previous research has explored associations between DXA parameters and mortality, fractures, falls, and quality of life [22,57,58].

MRI has the best image resolution and a high level of accuracy and is central to much research in brain-imaging studies. MRI utilises a strong magnetic field and radio waves to generate images. While MRI does not use X-rays or other ionising radiation, it is expensive and not widely available [55].

Peripheral quantitative computed tomography (pQCT) is used for making 3D measurements of bone, muscle and fat in peripheral sections of the body, such as the forearm and leg [55]. Fat area data provide the area from a cross-section of arms or legs obtained from a chosen percentage of landmark bones. The inter-muscular and intra-muscular adipose tissue can also be measured; muscle density represents a surrogate of fat infiltration to muscle. Previous research has detected associations between pQCT parameters and mortality, fractures, falls, and quality of life [59,60,61,62].

Air displacement plethysmography (Bod Pod), is a technique that rapidly estimates body fat percentage and is useful for assessing minor differences in body composition in overweight and obese individuals [63]. However, a recent study questioned the accuracy of the Bod Pod’s estimates of body fat percentage [64].

## 7. Conclusions and Unanswered Questions

The evidence to date suggests that obesity is associated with reduced cognitive function, plasticity and brain volumes, and altered brain structure. As parallel changes in body composition and the brain might be primarily driven by excessive accumulation of fat tissue, it is plausible that interventions that alter body composition might have the added benefit of modifying the trajectory of cognitive decline. Therefore, there is a need to investigate functional and structural changes in body composition and the brain, and the underlying mechanisms that might link these changes, in order to develop an evidence base that could be readily translated into interventions to improve both physical and cognitive health and, ultimately, delay the development of dementia.
